# The Power of Implicit Social Relation in Rating Prediction of Social Recommender Systems

**DOI:** 10.1371/journal.pone.0154848

**Published:** 2016-05-06

**Authors:** Waleed Reafee, Naomie Salim, Atif Khan

**Affiliations:** 1 Department of Computer Science, Faculty of Computing, Universiti Teknologi Malaysia, Skudai, Johor, Malaysia; 2 Department of Information Systems, Faculty of Computing, Universiti Teknologi Malaysia, Skudai, Johor, Malaysia; 3 Department of Mathematics and Computer Science, Faculty of Pure and Applied Science, International University of Africa, Khartoum, Sudan; Beijing University of Posts and Telecommunications, CHINA

## Abstract

The explosive growth of social networks in recent times has presented a powerful source of information to be utilized as an extra source for assisting in the social recommendation problems. The social recommendation methods that are based on probabilistic matrix factorization improved the recommendation accuracy and partly solved the cold-start and data sparsity problems. However, these methods only exploited the explicit social relations and almost completely ignored the implicit social relations. In this article, we firstly propose an algorithm to extract the implicit relation in the undirected graphs of social networks by exploiting the link prediction techniques. Furthermore, we propose a new probabilistic matrix factorization method to alleviate the data sparsity problem through incorporating explicit friendship and implicit friendship. We evaluate our proposed approach on two real datasets, Last.Fm and Douban. The experimental results show that our method performs much better than the state-of-the-art approaches, which indicates the importance of incorporating implicit social relations in the recommendation process to address the poor prediction accuracy.

## Introduction

Recommender systems (RS) have been widely studied and implemented in several directions, such as academia and industry. RS deal very well with information overload problems by suggesting to users the items that are potentially of interest to them, such as a product recommendation from Amazon. However, with the rapidly increasing number of users and items, the problem of data sparsity (the user rates a very few items from the total number of items in the database) has been increasingly intractable.

Traditional collaborative filtering (TCF) approaches mainly focus on utilizing users’ historical preferences on the purchased products to predict users’ interests [1, 2]. TCF is very successful in the real world, and is easy to understand and implement [1], but does not consider any additional information, making it difficult to solve the sparsity problem and provide accurate recommendations.

Due to the increasing growth of social networks in recent years and the large amount of information generated, it has become necessary for RS to exploit this information to improve recommendation accuracy. Several social recommendation methods have been proposed and have started to use social network information as additional input in the recommendation process [3–11]. However, data sparsity and poor prediction accuracy problems still exist.

In order to exploit the social network information in the best way, we refer to the users’ relationship according to two types, namely, explicit friendship and implicit friendship. An explicit friendship that is formed by users explicitly adds other individuals as ‘friends’, while an implicit friendship is a weighted graph, in which edge weights are determined by the frequency, interactions and common neighbors between users in social networks. For example, in social networks such as Facebook, user A can create a friendship relation with user B by sending a request for him/her and wait until he/she accepts. This explicit friendship relation between users A and B may only represent part of the user relationship based on the social connectivity between them. On the other hand, user A might have a high probability with user C to be linked based on the common neighbors ‘common friends’ in the social network, so many users may be related to each other through an implicit friendship relation.

Most of the existing social recommendation methods [5, 12] to the best of our knowledge, assume that the user preferences may be influenced by a number of explicit social friends. We believe that the social influence among people is not only determined by the social connectivity, but also by the amount of common neighbors between social network nodes and also the volume of interactions between them. Therefore, in this paper, by exploiting all the social friendship relation types, both explicit and implicit, and the available data for rating prediction, we can achieve better rating prediction accuracy.

The big challenge in this work is how to extract the implicit relationships between users when we only have the social network structure. However, the link prediction techniques are the most successful methods to play this role. Therefore, in this article, our interest is related to two main questions:

- How can we exploit the link prediction techniques in the undirected graph to extract the implicit relationship?
- Can the implicit relationship be helpful in overcoming the sparsity problem and improving the recommendation accuracy?

In this paper, three data sources, users-item rating, explicit social relation and implicit social relation, fuse together, based on probabilistic matrix factorization. We focus on extracting the hidden relation, which we call implicit friendship and combine it with explicit friendship.

The main contributions of this paper include the following: (1) we propose the “Possibility of Friendship between Non-Friends” (PFNF) algorithm to extract the implicit friendship based on the social network structure of an undirected graph. (2) Furthermore, we propose the explicit and implicit social relation probabilistic matrix factorization (EISR) model; in this method two social regularization terms are added to the objective function of our model to alleviate the data sparsity and poor prediction accuracy problems. The first term is the explicit social regularization, which is measured by the available user-user friend data, and assumes that the user preferences may be influenced by a number of explicit friends. The second implicit social regularization term is measured by the higher probability between two users based on common neighbors and assumes that the user preferences may be influenced by a number of implicit friends. (3) Finally, we conduct experiments on the two well-known real datasets, Last.fm [13] and Douban [5], which shows that our method of EISR outperforms the state-of-the-art collaborative filtering algorithms that do not use any social relation information, and the existing social recommendation algorithm that uses explicit social relation information. We also test our method under a data sparsity scenario and show that it is more effective than the diffusion-based method to overcome these problems. We believe the recommendation accuracy through our method will be improved by incorporating the explicit and implicit friendship relation into probabilistic matrix factorization.

The rest of this paper is organized as follows: we summarize the related work and background of social network analysis and major methods based on a matrix factorization for social RS in Section 1. In Section 2, we describe and briefly introduce a review of the material and methods that we study in this paper. The results and discussion are presented in Section 3 followed by the conclusion in Section 4.

## Theoretical Background

Our work is related to two research fields; social network analysis and RS. Therefore, we review the link prediction techniques of social networks. Furthermore, we review the approaches of RS, including TCF and social-based RS.

### 1.1 Link Prediction in Social Networks

With the explosion of Web 2.0 applications, such as forums, blogs, social networks, social bookmarking and several other types of social media, the analysis of networks has been the direction of many research studies. Link prediction, which refers to the task of predicting the edges that are currently missing, but will be added to the network in the future based on past snapshots of the network, has become one of the most important problems in the area of network analysis. Recently, there have been many investigations on network analysis, and the link prediction has been studied with various social networks such as biological interaction networks [14, 15], scientific co-authorship networks [16] and with e-commerce-like recommendation systems [17, 18]. Various relational learning methods have been proposed for the link prediction problem, typically exploiting both the link structure itself and the rich descriptive attributes of the data objects [19]. These methods can be divided into three types: link prediction based on features, link prediction based on structure and a hybrid of the first two types.

The methods of link prediction based on features takes into account the information content of the interactions between the nodes or their attributes and do not adopt any graph theory. Link prediction based on structure typically only uses the graph structure, the nodes and the edges to infer the future links. Many researchers only adopted the graph structure for link prediction problems [20–22]. Liben-Nowell and Kleinberg in [20] presented a comparative study of link prediction based on structure methods, studying the power of a topological graph by testing it on bibliographic datasets. For example, the probabilistic evaluation model proposed by Kashima and Abe [21] based on network structures, used a biological network dataset and the model appeared efficient compared with the link prediction methods based on the various topological networks. In 2006, Hasan et al.[22] extended their work and showed that the graph structure of social network acts as a good source of information for the prediction of new friendship links between the nodes. Some studies of the link prediction problem proposed hybrid methods by using a combination of both link prediction based on the structure and features methods, such as [23–25]; all of them focused on integrating the network structure and node attributes, and showed improved performance in the link prediction; however, the problem is that the node attributes are not always available.

### 1.2 Traditional Collaborative Filtering

Traditional collaborative filtering (TCF) is the most popular recommendation technique and is widely adopted in many commercial domains including businesses, such as Amazon [26], TiVo and Yahoo! [27], while, in [28], the GroupLens system used TCF for NetNews. The TCF methods are mainly divided into two main categories: memory-based [29–31] and model-based [32–34] methods.

The memory-based approaches (also known as neighborhood-based approaches) focus mainly on finding the similarity between users or items. Memory-based approaches can be further classified as ‘user-based approaches’ [29, 35, 36] and ‘item-based approaches’ [30, 37] to predict the recommendation of active users.

The model-based approaches are used to train a predefined model and then use a model to predict the recommendation, in other words, model-based algorithms aim to find behavioral patterns between users through reliance on data mining or machine learning techniques. Several models have been proposed to be used in model-based approaches, such as Bayesian models [38], latent semantic models [32] and clustering models [33, 39]. The big difference between the two types of memory-based and model-based algorithms according to their method of processing data, is that the model-based estimates the ratings by utilizing machine learning techniques and statistics to learn about a model from the basic data, while the memory-based approach uses some heuristic rules to predict the ratings [40].

However, the traditional collaborative filtering techniques only utilize user-item interaction and those methods that depend on memory-based approaches still suffers from the cold-start and data sparsity problems, and have difficulty in dealing with large datasets.

### 1.3 Social Recommendation Methods

In this article we look at the concept of social RS based on social friendship relations between users in social networks, in which the relationship is exchanged between the parties in the social network. This represents how the concerned users indulge in mutual interaction on social networks; unlike the trust relationship that can be one-sided with different trust values. The social recommendation methods, especially those based on matrix factorization, which have drawn a lot of attention recently due to their efficiency in dealing with large datasets, will be reviewed.

There have been a few related works recently that deal with the social recommendation problems, and some methods have been proposed that incorporate the social information into probabilistic matrix factorization [5, 7, 9, 12, 41].

[5] proposed two social recommendation algorithms, SR1 and SR2 based on the similarity between users, which impose social regularization terms to constrain matrix factorization. The results show that users’ explicit friendship information can help to improve the prediction accuracy of RS. Zhang et al. [9], based on the approaches in [5], proposed a method to deal with the cold-start scenario and improve the prediction accuracy. In [12], to be effective, the authors proposed a regularization-based method and used social network information in matrix factorization. They defined two social regularization terms, the friendship between users and the correlation between the item and user, which they fused into the matrix factorization objective function. Huang et al.[7], based on the concept of the graph model theory, proposed a semi-supervised probabilistic model to address the sparsity problem in which they combined the user preference and the influence of the user’s friends and they proposed a general model. Based on the method proposed by Ma et al. [5], the authors in [41] proposed a similar approach to formula (10), but based on assumption that we don't have any additional information such as ‘explicit friendship’ and then presented a comparative study of the role of social relations. It showed modest improvement over matrix factorization on several movie recommendation datasets.

We note that Ma et al. proposed the three classic approaches, namely, RSTE [3], SoReg [42] and SoRec [4] by incorporating the social network information into the PMF model. These approaches provided the impact of social relation for improve the recommendation accuracy. Recently some of the latest expansion of PMF to benefit from social networks information it have been proposed such as, [43] and [44].

However, the social RS problem has not been studied very well in previous work. Therefore, there are certain drawbacks among the previous studies. In the method proposed in [5] the authors worked based on the assumption that the user preferences may only be influenced by explicit friends. This assumption does not reflect the growth that occurs in the social network. In the approach proposed in [41], the social relationship information was not fully considered, and, essentially this approach suffers from the limitation that it cannot fuse the explicit and implicit friendship relation, with the author just utilizing the user-item rating to measure the similarity between users. Zhang et al. proposed a social recommendation method in [9], which the authors utilize as trust network information in the experimental process; simply, the trust relation always appears unidirectional, which is contrary to the friendship relation in the social recommendation methods. Huang et al. [7] and [12], only used the social context information, such as tagging and did not incorporate the situation of implicit friendship between users.

In contrast to the incorporation of the explicit friendship relation, unfortunately the majority of the literature on social recommendation totally ignores the role of the implicit friendship relation in boosting the accuracy of the recommendations.

In this paper, our method is a fully social recommendation method and differs from the previous work because we focus on the leverage of the hidden social relations between users. Accordingly we have investigated the power of link prediction techniques to extract the implicit friendship and incorporate it with explicit friendship into probabilistic matrix factorization to improve the recommendation accuracy.

## Materials and Methods

### 2.1 Problem Definition

**Fig 1:**
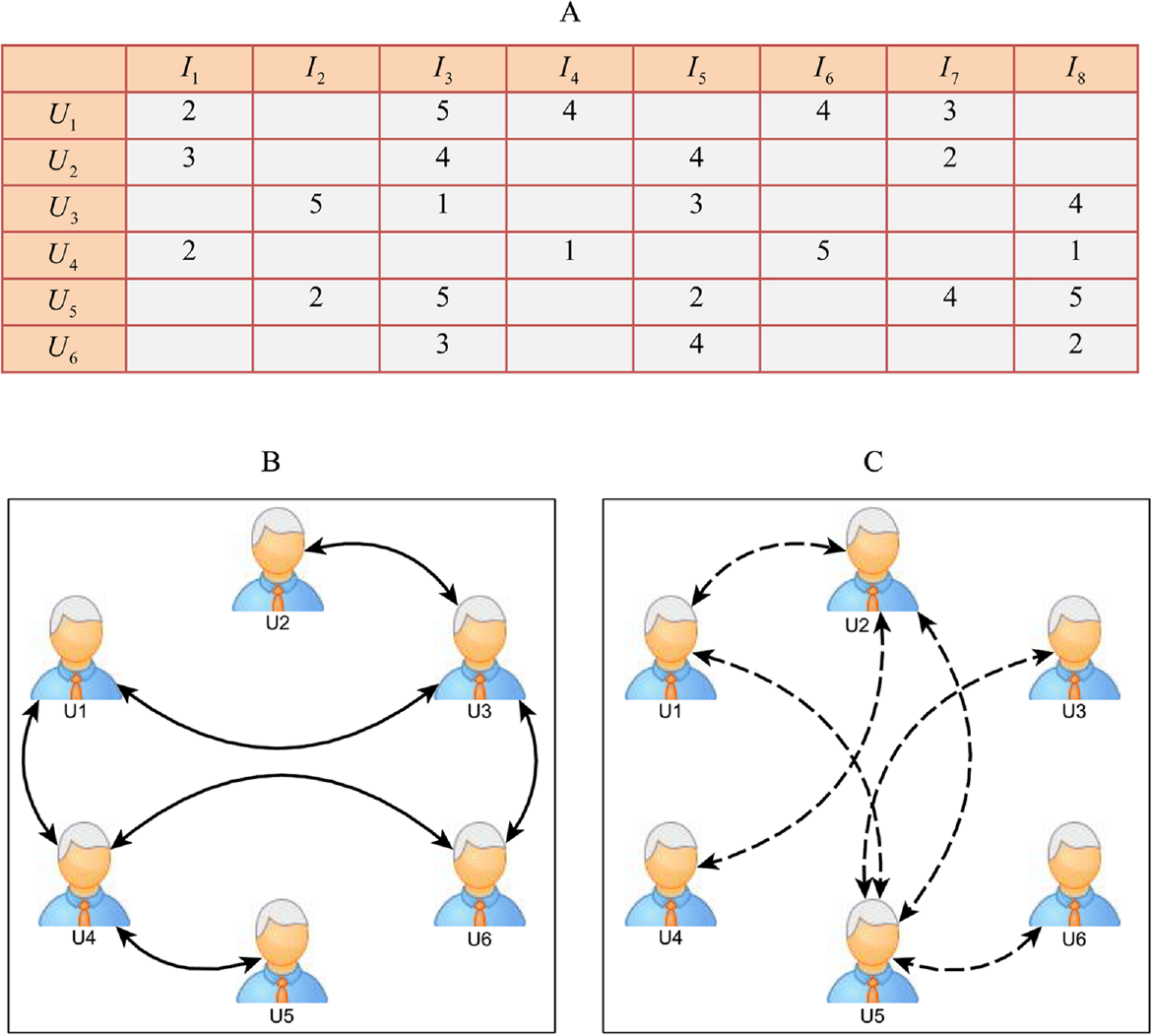
Illustration of the three main data sources in our recommendation method.

We begin by providing a definition of the problem under investigation. The problem we investigate in this paper considers three major data sources:

(1) the user-item rating matrix of *m* users and *n* items is denoted as an *m* × *n* matrix *R* where *R*_*ij*_ (*i* = 1……….*m*; *j* = 1……….*n*) represents the rating of the user *i* for the item *j*; (2) the explicit relation of social network *G*1 = (*V*, *E*1), where (*i*, *f*) ∈ *E*1 indicates that users *i* and *f* are explicitly friends; (3) the implicit relation of social network *G*2 = (*V*, *E*2), where (*i*, *h*) ∈ *E*2 indicates users *i* and *h* are implicitly friends. The illustration of our problem data sources is shown in Fig 1; Fig 1A shows the user-item rating matrix *R*, Fig 1B illustrates the explicit friends’ network and Fig 1C shows the implicit friends’ network.

Accordingly, the problem we investigate in this paper is how to extract and employ the different types of friendship relations to predict the missing values from the user-item rating matrix.

### 2.2 Probabilistic Matrix Factorization

Before we introduce the proposed methods, we first give a brief review of the probabilistic matrix factorization methods (PMF) that work to identify something hidden under the data. PMF is usually more effective in the RS field because it allows one to discover the latent features underlying the interaction between users and items.

**Fig 2:**
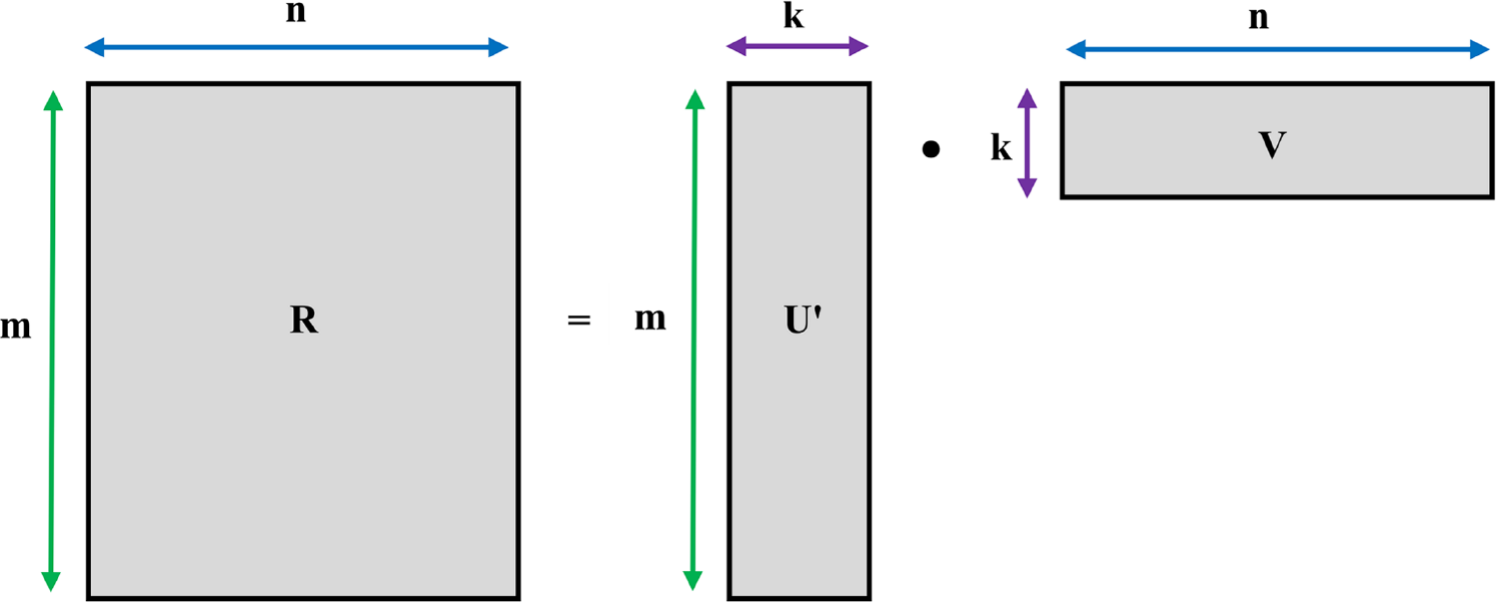
Approximate rating matrix *R* via user and item feature vectors.

Using *m* set of users, *n* set of items, and *R* as the rating matrix of size *m* × *n*, the PMF method looks up to approximate the rating matrix *R* via a multiplication of *k*-rank factors. The latent users and item factors are denoted as *U* ∈ *R*^*k*×*n*^ and *V* ∈ *R*^*k*×*m*^, respectively, with the dimension of user feature vector and item feature vector denoted as *k*, which is much less than *m* and *n* as illustrated in Fig 2.

In the PMF [45], the conditional probability over the observed rating can thus be formulated as:

$$p(R|U,V,\sigma_{R}^{2})={\prod_{i=1}^{m}\prod_{j=1}^{n}\left[N\left(R_{ij}|g(U_{i}^{T}V_{j}),\sigma_{R}^{2}\right)\right]}^{I_{ij}^{R}},$$ (1)

Where *N*(*x* | *μ*, *σ*^2^) represents the probability density function of the Gaussian distribution with mean *μ* and variance *σ*^2^, $I_{ij}^{R}$ is the indicator function that is equal to 1 if user *i* rated item *j* and equal to 0 otherwise. The function *g*(*x*) is the logistic function *g*(*x*) = 1/(1 + exp(−*x*)) which makes it possible to bound the range of $U_{i}^{T}V_{j}$ within the range [0, 1]. The zero-mean spherical Gaussian priors are placed on the user and item feature vectors:

$$p(U|\sigma_{U}^{2})=\prod_{i=1}^{m}N\left(U_{i}|0,\sigma_{U}^{2}I\right),$$ (2)

$$p(V|\sigma_{V}^{2})=\prod_{j=1}^{n}N\left(V_{j}|0,\;\sigma_{V}^{2}I\right).$$ (3)

Hence, through a Bayesian inference, we maximize the posterior distribution by minimizing the sum-of-squares of factorization error with quadratic regularization terms:

$$E=\frac{1}{2}\sum_{i=1}^{m}\sum_{j=1}^{n}I_{ij}{\left(R_{ij}-g(U_{i}^{T}V_{j})\right)}^{2}+\frac{\lambda_{U}}{2}\sum_{i=1}^{m}U_{i}^{T}U_{i}+\frac{\lambda_{V}}{2}\sum_{j=1}^{n}V_{j}^{T}V_{j}$$ (4)

Where $\lambda_{U}=\sigma_{R}^{2}/\sigma_{U}^{2}$ and $\lambda_{V}=\sigma_{R}^{2}/\sigma_{V}^{2}$. The local minimum of formula (4) is found through the gradient descent method.

PMF is the most popular method in RS due to its efficiency in dealing with large datasets and its success in terms of the speed-accuracy trade-off in the Netflix Prize competition. Although it has drawbacks, PMF assumes that user vectors and item vectors are independently and identically distributed and, in general, ignores the additional data sources such as social network information. We believe that the social network information, particularly the social relations among users, can alleviate the data sparsity problem and improve recommendation accuracy.

### 2.3 Social Regularization

In order to model the social recommendation problem more accurately, [5] proposed two social regularization (SR) models; average-based regularization and individual-based regularization. They are general social recommendation models that, typically, have added a SR term to the objective function and minimized it to get more accurate recommendation results than NMF [46], PMF [45], RSTE [3] and other state-of-art models on large real datasets. In this paper, we only consider the individual-based regularization model.

The objective function of this model is formulated as:

$$E=\frac{1}{2}\sum_{i=1}^{m}\sum_{j=1}^{n}I_{ij}{\left(R_{ij}-U_{i}^{T}V_{j}\right)}^{2}+\frac{\lambda_{U}}{2}{\left\|U\right\|}_{Fro}^{2}+\frac{\lambda_{V}}{2}{\left\|V\right\|}_{Fro}^{2}+\frac{\beta_{E}}{2}\sum_{i=1}^{m}\sum_{f\in F(i)}sim(i,f){\left\|U_{i}-U_{f}\right\|}_{Fro}^{2}$$ (5)

Where *β*_*E*_ > 0 is the parameter to control the strength of the explicit relationship as SR, *sim*(*i*, *f*) is the similarity function to measure the similarity between user *i* and his/her explicit friends, *F*(*i*) is the set of explicit friends of user *i*, and ${\left\|.\right\|}_{Fro}^{2}$ denotes the Frobenius norm.

In the SR social recommendation method, the social relationship between users is usually established through adding each other explicitly as friends, where the second term in the model formula (5) is SR in which it is assumed that the user preferences may be influenced by a number of explicit friends. According to this, it works to minimize the different feature vectors of user *i* and his/her friends. This assumption is incomplete and unfair, because, in the real world, some of our explicit friends may have different tastes, also the social network grows dramatically and the hidden friendship relation can be created in the near future and may affect the user's preferences.

Clearly, the SR method just cares about users’ explicit social relations and has largely ignored the implicit social relations that can be established between users in social networks. Therefore, if we extracted the implicit social relations among users by adopting the link prediction methods, we believe the recommendation accuracy will be improved through incorporating the explicit and implicit social relations into PMF. Based on this idea, we propose our method.

### 2.4 Proposed Method

In this section, we present our proposed explicit and implicit social relation (EISR) model for social recommendation.

In our model, and based on the fact that the friend of a friend can become a friend, we assume that users with many friends pay more attention to creating new friendship relations in the future and the preferences of that user may be affected by the preferences of his/her explicit and implicit friends. Therefore, we propose a Possibility of Friendship between Non-Friends (PFNF) algorithm to extract the implicit friendship and incorporate both explicit and implicit friendship into the proposed model (EISR). In the sections below, we introduce the (PFNF) algorithm and our proposed model (EISR) in more detail.

#### 2.4.1 Possibility of Friendship Between Non-Friends

In this section, we review the proposed algorithm “Possibility of Friendship between Non-Friends” (PFNF) to extract the hidden friendship on undirected graphs. We aim to predict the implicit relation between users even if they have no explicit relation before, based on the link prediction methods.

Suppose we have an undirected graph *G* = (*V*, *E*) that represents the topological structure of the social network, where the vertex set *V* represents all the users in a social network and the edge set *E* represents the explicit relation between users denoted by *e* = (*x*, *y*) ∈ *E* where *x*, *y* ∈ *V*. Our goal is to extract the implicit relation hidden behind the social network structure, so that for each two vertices (nodes) *x*, *y* ∈ *V* we can predict the probability between them to be linked by using the link prediction methods that are usually exploited in the social network analysis. We use the link prediction methods (Jaccard coefficient [47], Common neighbors [16], Preferential attachment [48] and Resource allocation index [49]) which are based on node neighborhoods. If we take here the neighbors in an undirected graph *G* as a feature for two users *x* and *y*, this approach leads to measure the similarity as follows:

Jaccard coefficient:

Sim(x,y)=|Γ(x)∩Γ(y)|/|Γ(x)∪Γ(y)| (6)

Common neighbors:

$$Sim(x,y)=\frac{1}{k}(\left|\Gamma (x)\;\cap \;\Gamma (y)\right|)$$ (7)

Preferential attachment:

$$Sim(x,y)=\frac{1}{k}(\left|\Gamma (x)\right|•\left|\Gamma (y)\right|)$$ (8)

Resource allocation index:

$$Sim(x,y)=\sum_{z\in \left|\Gamma (x)\;\cap \;\Gamma (y)\right|}\frac{1}{\left|\Gamma (z)\right|}$$ (9)

Where for a node *x* and *y*, let Γ(*x*) and Γ(*y*) denote the set of neighbors of *x* and *y* respectively, in undirected graph *G* and *k* is a normalization factor. The implicit friendship relation can be calculated from the output of the algorithm as follows:

Algorithm 1. Computing of PFNF

1. Input: undirected matrix *M*, the number of users *U*, the probability of choosing *P*

2. Output: implicit social relations matrix *N*

3. for *u* = 1:*U* do

4. Determine the friends *F* of user *u*

5. Determine the non-friends *NF* of user *u*

6. end for

7. for *u* = 1:*U* do

8. Calculate the similarity *S* between *u* and *NF* according to formulas (6) or (7) or (8) or (9)

9. if *S*>*P* then

10. Choose *u* and *NF* to the matrix *N*

11. end if

12. end for

13. return *N*

#### 2.4.2 Explicit and Implicit Social Relation Probabilistic Matrix Factorization (EISR)

As already reviewed, most of the literature reviewed on social recommendation has primarily focused on the explicit social relation and ignored the implicit social relation among the users in the social network. Therefore, we focus on developing the EISR model based on matrix factorization by utilizing both the explicit and implicit friendship relation.

In section (2.2), based on one data source giving the user-item rating matrix, the observed rating *R*_*ij*_ is interpreted by the user *i* preference on item *j*, while in section (2.3), based on two data sources given the user-item rating matrix and user's social relation, the observed rating *R*_*ij*_ is realized as the preference of user *i* on item *j* based on explicit friends’ preferences. Hence, in order to define our model more flexibly, we use three data sources by considering the power of the implicit social relation. The objective function of formula (5) should be modified by incorporating explicit SR and implicit SR into PMF. We obtain the new objective function as below:

$$\begin{array}{l} E=\frac{1}{2}\sum_{i=1}^{m}\sum_{j=1}^{n}I_{ij}{\left(R_{ij}-g\left(U_{i}^{T}V_{j}\right)\right)}^{2}+\frac{\lambda_{U}}{2}{\left\|U\right\|}_{Fro}^{2}+\frac{\lambda_{V}}{2}{\left\|V\right\|}_{Fro}^{2}\\ \;+\frac{\beta_{E}}{2}\sum_{i=1}^{m}\sum_{f\in F\;(i)}sim(i,f){\left\|U_{i}-U_{f}\right\|}_{Fro}^{2}+\frac{\beta_{I}}{2}\sum_{i=1}^{m}\sum_{f^{*}\in F^{*}(i)}sim(i,f^{*}){\left\|U_{i}-U_{f^{*}}\right\|}_{Fro}^{2} \end{array}$$ (10)

Where *β*_*E*_, *β*_*I*_ > 0 are the parameters to control the strength of the explicit and implicit relations as SRs, respectively, *sim*(*i*, *f*), *sim*(*i*, f*) are the similarity functions to measure the similarity between user *i* and his/her explicit and implicit friends respectively, *F*(*i*) is the set of explicit friends of a user *i*, F*(*i*) is the set of implicit friends of a user *i*, and ${\left\|.\right\|}_{Fro}^{2}$ denotes the Frobenius norm. The function *g*(*x*) is the logistic function *g*(*x*) = 1/(1 + exp(−*x*)), which makes it possible to bound the range of $U_{i}^{T}V_{j}$ within the range [0, 1].

We can find a local minimum of the objective function of formula (10) by performing a gradient descent on *U*_*i*_ and *V*_*j*_ for all user *i* and item *j*. However, due to the huge number of preference pairs, it is expensive to update the latent features over all pairs. We adopt a mini-batch gradient descent algorithm, and update the latent factors by the following gradients:

$$\begin{array}{l} \frac{\partial E}{\partial U_{i}}=\sum_{j=1}^{n}I_{ij}(g(U_{i}^{T}V_{j})-R_{ij})g^{\prime }(U_{i}^{T}V_{j})V_{j}+\lambda_{U}U_{i}\\ \;+\beta_{E}\sum_{f\in F^{+}\left(i\right)}sim(i,f)(U_{i}-U_{f})+\beta_{E}\sum_{g\in F^{-}\left(i\right)}sim(i,g)(U_{i}-U_{g})\\ \;+\beta_{I}\sum_{f^{*}\in F^{+*}\left(i\right)}sim(i,f^{*})(U_{i}-U_{f^{*}})+\beta_{I}\sum_{f^{*}\in F^{-*}\left(i\right)}sim(i,g^{*})(U_{i}-U_{g^{*}}) \end{array}$$ (11)

We represent the relationship between the users through an undirected graph in section (2.4.1). So, in formula (11), the out-link explicit friends equals the in-link explicit friends *F*^+^(*i*) = *F*^−^(*i*) for each user and the out-link implicit friends equals the in-link implicit friends *F*^+^*(*i*) = *F*^−^*(*i*) for each user. We can rewrite the formula (11) as follows:

$$\begin{array}{l} \frac{\partial E}{\partial U_{i}}=\sum_{j=1}^{n}I_{ij}(g(U_{i}^{T}V_{j})-R_{ij})g^{\prime }(U_{i}^{T}V_{j})V_{j}+\lambda_{U}U_{i}\\ \;+\beta_{E}\sum_{f\in F\left(i\right)}sim(i,f)(U_{i}-U_{f})+\beta_{I}\sum_{f^{*}\in F^{*}\left(i\right)}sim(i,f^{*})(U_{i}-U_{f^{*}}) \end{array}$$ (12)

$$\frac{\partial E}{\partial V_{j}}=\sum_{i=1}^{m}I_{ij}(g(U_{i}^{T}V_{j})-R_{ij})g^{\prime }(U_{i}^{T}V_{j})U_{i}+\lambda_{V}V_{j}$$ (13)

Where *g*′(*x*) = exp(*x*)/(1 + exp(−*x*))^2^ is the derivative of the logistic function *g*(*x*). The process of estimating the latent low-rank matrices *U*_*i*_ and *V*_*j*_ are described in algorithm 2.

Algorithm 2. Learning of EISR

1. Input: observed rating matrix *R*, explicit relationship *X*, implicit relationship *P*, learning rate *η*, max iteration *T*, mini-batch size *M*, number of batches *S*

2. Output: user and item latent factors *U*,*V*

3. Initialize latent factors: *U*,*V* ∼ *N*(*x*|0,*I*) 

4. for *t* = 1:*T* do

5. for *s* = 1:*S* do

6. (*i*, *j*) sample random from *R* size *M*

7. Calculate the gradient ∂*R*/∂*U*_*i*_ according to formula (12) and using *R*,*U*,*V*,*X*,*P*

8. Calculate the gradient ∂*R*/∂*V*_*j*_ according to formula (13) and using *R*,*U*,*V*

9. Update: *U*_*i*_ = *U*_*i*_ − *η*(∂*R*/∂*U*_*i*_)

10. Update: *V*_*j*_ = *V*_*j*_−*η*(∂*R*/∂*V*_*j*_)

11. end for

12. end for

13. return *U*,*V*

### 2.5 Data and Metrics

In this section, we begin by introducing the data sets we use in our experiments, followed by the metrics we employ to evaluate the results.

Dataset: several datasets have been widely used to evaluate the performance of recommendation methods, such as Netflix, Movielens, Douban, Last.fm etc,. However, only Douban and Last.fm contain social network information. Hence, we choose them to evaluate our proposed method (datasets are free to download as S1 Data and S2 Data).

Last.fm dataset was released in the framework of the 2nd International Workshop on Information Heterogeneity and Fusion in Recommender Systems (HetRec 2011) [13]. Last.fm is a popular online music system (http://www.last.fm) founded in the United Kingdom in 2002. It has claimed over 40 million active users based in more than 190 countries. Last.fm allows users to listen to several types of music online in the system and allows users to establish friendship relations with other users. The Last.fm dataset only records the listening of each user for particular artists. Therefore, for the purpose of effective testing, we use similar way to [6] to map the listening counts into integer values of 1 to 5 to represent the extent of favor of the artists. The mapping formula is given as:

$$r=\{\begin{array}{l} \;\left\lfloor {\text{log}}_{10}\;l\right\rfloor +1\;,\;if\left\lfloor {\text{log}}_{10}\;l\right\rfloor +1\leq 5\\ \;5\;,\;otherwise \end{array}$$ (14)

Where r is the mapped value, l is the listening count and ⌊g⌋ is the operator of rounding towards zero.

Since the original Last.fm dataset is large, we construct a good subset by randomly choosing some users who have at least five friends for Last.fm and took all the movies they had rated. To conduct the coming experiments, we sample a subset of the dataset with *n* = 1,123 users and *m* = 18744 different items, respectively. The total number of observed ratings in the sampled datasets is *r* = 55,140.

The Douban dataset is crawled by Ma Hao [5]. Douban is a Chinese social website launched on March 6, 2005, providing user rating, review and recommendation services for movies, books and music. It provides Facebook-like social networking services where users can make friends with each other through the email communication. The rating values in the Douban dataset are discreet values ranging from not helpful (1/5) to most helpful (5/5). We sample a subset of the dataset with *n* = 1,384 users and *m* = 58,402 different items, respectively. The total number of observed ratings in the sampled dataset is *r* = 411,623. The basic statistics of the datasets are shown in Table 1.

**Table 1:** Statistics of sample data from the Last.fm and Douban datasets.

Datasets	Num. of users	Num. of items	Num. of ratings	Sparsity	Num. of explicit relation	Num. of implicit relation
Last.fm	1123	18744	55140	2.619 × 10^−3^	11064	166568
Douban	1384	58402	411623	5.092 × 10^−3^	25361	597839

The sparsity is obtained by *r*/ (*n* × *m*), where n, m and r are the number of users, items, and ratings respectively.

Evaluation metrics: we choose two most popular metrics that are well-known in the literature of RS [50], the Mean Absolute Error (MAE) and the Root Mean Square Error (RMSE), to measure the prediction accuracy of our proposed model in comparison with other recommendation methods.

The MAE metric is defined as:

$$MAE=\frac{1}{T}\sum_{i,j}\left|R_{ij}-{\hat{R}}_{ij}\right|$$ (15)

The RMSE metric is defined as:

$$RMSE=\sqrt{\frac{1}{T}\sum_{i,j}{\left(R_{ij}-{\hat{R}}_{ij}\right)}^{2}}$$ (16)

Where *R*_*ij*_ denotes the rating user *i* gave to item *j*, ${\hat{R}}_{ij}$ denotes the rating user *i* gave to item *j* as predicted by a method, and *T* denotes the total number of tested ratings.

### 2.6 Experimental Design

In this section, to better evaluate the effect of utilizing the social relation information on predicting recommendations, our aim here is to prepare the parameter values and experimental settings for implementing our method and all the compared methods.

Evaluate the prediction: we conduct experiments on the two real datasets Last.fm and Douban to validate the effectiveness of our proposed method. We employ different amounts of ratings as training data (90%, 80% and 60%), and use the rest of the ratings as testing data. Training data 80%, for example, means we randomly select 80% of the ratings from the user-item rating matrix as the training data to predict the remaining 20% of the ratings. We implement our proposed method in Matlab R2013a, and use the Gephi graph visualization software to depict the PFNF result. All the experiments are conducted on Windows Vista with Intel Xeon processors (E5420 @ 2.5 GHz 2.5 GHz) and 12 GB memory.

Comparative algorithms: in order to show the performance improvement of our recommendation algorithm with explicit and implicit social relations, we note that there is no algorithm in the literature that exploits both explicit and implicit friendship together in factorization steps. We choose the following state-of-the-art methods for comparison.

- PMF: We compare our method with the baseline method PMF proposed by Salakhutdinov et al. [45], which only uses the user-item rating matrix for recommendation without consideration of any social factors.
- SR: This method is proposed by Ma et al. [5], SR is social recommendation algorithm formulated in the optimization problem in formula (5), which uses the social information for predicting the missing value.
- HeatS: This method is represented by Zhou et al [51]. To facilitate comparison, we refer this method as HeatS. HeatS is a diffusion-based recommendation method, which exploits the heat-spreading algorithm to address the challenge of diversity.
- HP: This method is Hybrid of Heats and Probs methods which is the stat-of-the-art diffusion-based methods represented by Zhou et al [51]. we refer this method as HP.

Parameter setting: we depict the meanings and settings of all parameters of our experiments and tune the parameters of our method and all compared methods to reach their best performance.

- *K*: The dimension of the latent features. Here we set *K* = 50 on the Last.fm and Douban datasets.
- *λ*_*U*_, *λ*_*V*_: Two regularization parameters in the second and third term of the objective function (10), respectively. Here we set *λ*_*U*_, *λ*_*V*_ = 0.01 on the Last.fm dataset and *λ*_*U*_, *λ*_*V*_ = 0.001 on the Douban dataset.
- *β*_*E*_: The weight of explicit friendship parameter in the fourth term of the objective function (10). We set the parameter *β*_*E*_ = 0.0002 on the Last.fm dataset and *β*_*E*_ = 0.001. on the Douban dataset.
- *β*_*I*_: The weight of implicit friendship parameter in the fifth term of the objective function (10). We set the parameter *β*_*I*_ = 0.0002 on the Last.fm dataset and *β*_*I*_ = 0.001. on the Douban dataset.

## Results and Discussion

In this section, we will discuss the performance of our proposed method EISR compared with other state-of-the-art methods on the Last.fm and Douban datasets for ratings’ predictions. The random selection from the datasets was carried out five times independently and showed average results. We evaluate all the methods by both MAE and RMSE measures. There are in total four versions of our proposed method EISR by using the most common link prediction methods, EISR-Jaccard coefficient (EISR-JC), EISR-Common neighbors (EISR-CN), EISR-Preferential attachment (EISR-PA) and EISR-Resource allocation index (EISR-RAI).

According to the result in Tables 2 and 3, it can be observed that our method EISR-RAI outperforms the other EISR versions, (EISR-JC, EISR-CN and EISR-PA) on Last.fm and Douban datasets in the term of improved MAE and RMSE. For example, when the Resource allocation index Method (EISR-RAI) is used, the corresponding MAE is 0.4084 and RMSE is 0.5201 are more accurate than 0.4108 and 0.5221 related to MAE and RMSE respectively to the EISR version (JC-EISR) in the case of the 80% training data of Last.fm. We can mention that our method EISR that used the link prediction method RAI highly improves the prediction of recommendation, due to the RAI extracting the new hidden implicit relations more than the remaining link prediction methods. At the same time, the other EISR versions outperform all the recommendation methods. Indeed, this is due to the novel way that we use to fuse the old and new links in our methods. As a result, below, we only report the result of the best one (EISR-RAI) compared with PMF, SR and diffusion-based methods (HeatS and HP).

**Table 2:** The performance comparison based on Last.fm dataset.

Training	Metrics	HeatS	PMF (baseline)	HP	SR	EISR-JC	EISR-CN	EISR-PA	EISR-RAI
60%	MAE	0.4474	0.4387	0.4386	0.4321	0.4279	0.4259	0.4251	0.4223
60%	RMSE	0.5601	0.5482	0.5480	0.5394	0.5375	0.5355	0.5350	0.5342
80%	MAE	0.4310	0.4253	0.4251	0.4214	0.4108	0.4068	0.4065	0.4048
80%	RMSE	0.5491	0.5339	0.5337	0.5300	0.5221	0.5210	0.5209	0.5201
90%	MAE	0.4229	0.4189	0.4188	0.4137	0.4025	0.4017	0.4015	0.4001
90%	RMSE	0.5370	0.5230	0.5227	0.5212	0.5140	0.5120	0.5120	0.5110

**Table 3:** The performance comparison based on Douban dataset.

Training	Metrics	HeatS	PMF (baseline)	HP	SR	EISR (JC)	EISR (CN)	EISR (PA)	EISR (RAI)
60%	MAE	0.6482	0.6378	0.6376	0.6282	0.6168	0.6084	0.5985	0.5927
60%	RMSE	0.8024	0.7912	0.7911	0.7802	0.7681	0.7625	0.7558	0.7515
80%	MAE	0.6370	0.6277	0.6274	0.6093	0.5937	0.5898	0.5783	0.5730
80%	RMSE	0.7881	0.7794	0.7792	0.7615	0.7590	0.7545	0.7451	0.7435
90%	MAE	0.6234	0.6163	0.6161	0.5983	0.5883	0.5862	0.5783	0.5741
90%	RMSE	0.7764	0.7601	0.7600	0.7588	0.7501	0.7456	0.7386	0.7366

Looking at the results reported in Tables 2 and 3, we can see that our method consistently outperforms the other methods and obtains a lower MAE and RMSE than the PMF, SR, HeatS and HP under all settings of both datasets in the cases of (90%, 80% and 60%) training data. First, our proposed method EISR is more accurate than the PMF and diffusion-based methods (HeatS and HP) under the experimental design above, indicating that it is effective for incorporating the social network information in the recommendation. On the other side the hybrid diffusion-based method (HP) it appears more accurate than the individual diffusion-based method (HeatS) and showed modest improvement over PMF. Second, the EISR method outperforms the other social recommendation algorithm SR, as can be seen from Tables 2 and 3; incorporating explicit and implicit friendship improves the result more than just utilizing the explicit friendship, indicating that it is effective by incorporating the implicit friendship information in the recommendation. This suggests that when the user has a large number of friends, they tend to be more influenced by his/her friends’ interests and that a large network of friends is more effective in improving the recommendation accuracy. For example, on average, compared with SR, EISR improves the predictive accuracy by (3.4% relative to MAE, 1.2% relative to RMSE on Last.fm dataset and by 4.2% relative to MAE, 3% relative to RMSE on Douban dataset) in the cases of 90% training data of both datasets. And the improvement even exists when less training data is used in the cases of 80% and 60% training data on Last.fm and Douban datasets. This suggests that our proposed method is dealing very well with addressing the data sparsity problem.

### 3.1 The Impact of Utilizing PFNF Algorithm

**Fig 3:**
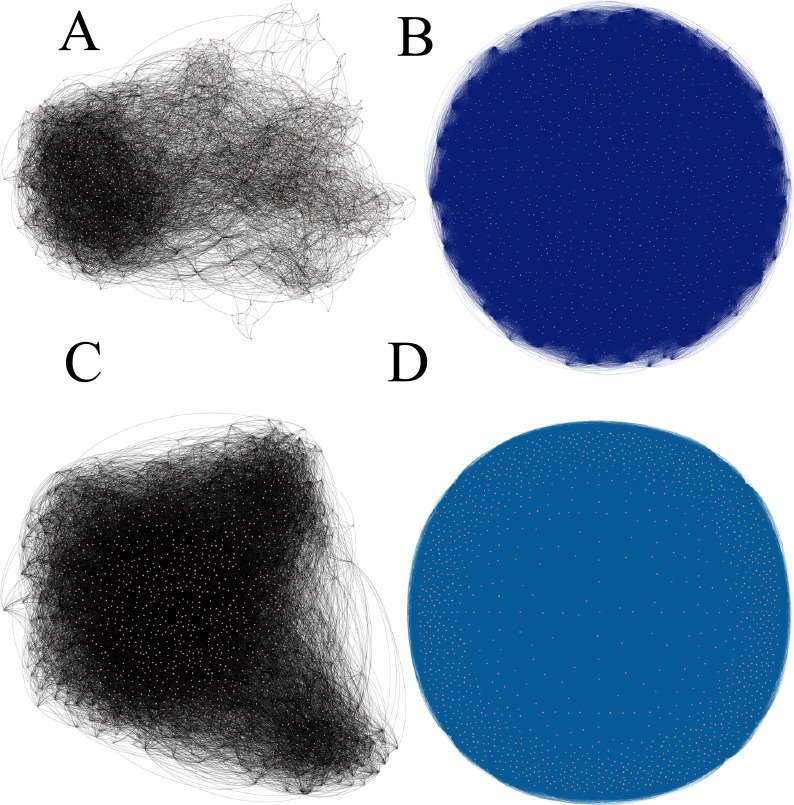
Result of PFNF discovering hidden friendship in a social network of Last.fm and Douban datasets based on RAI similarity metrics. A and C represent the user with his/her old explicit friends; B and D represent the same user with his/her new implicit friends.

We now turn to investigating the effect of utilizing a PFNF algorithm to extract the hidden friendship. In other words, we would like to experimentally evaluate whether our algorithm increases the social network density. We begin by showing the graphical result of the PFNF algorithm. This algorithm reveals the hidden structure in the social network, in which each node represents a user and each link represents a social relation between two users. Fig 3 depicts the PFNF output graph. Fig 3A and 3C are the synthetic social network containing a set of users and explicit friendship between them before we used the PFNF algorithm. In Fig 3B and 3D, after applying a PFNF algorithm, we discover strong links of implicit friendship between users that were not linked directly before that. For example, in comparing Fig 3A, 3B, 3C and 3D, we observe the PFNF algorithm improves the network density by discovering a new link in a social network and the number of links increases thanks to the application of link prediction methods. Tables 4 and 5 show the statistical results of using the PFNF algorithm depending on the link prediction methods. We can see that a large number of new links can be discovered when we used the Resource allocation index similarity metric.

**Table 4:** The basic statistics of using a PFNF algorithm on Last.fm dataset.

Matrics	Before using PFNF algorithm	After using a PFNF algorithm			
		JC	CN	PA	RAI
Graph type	Undirected	Undirected	Undirected	Undirected	Undirected
No. of users (nodes)	1123	1123	1123	1123	1123
No. of relations (links)	11063	150322	153341	153442	166568
Network density	0.018	0.239	0.243	0.244	0.264
Average degree	19.703	267.715	273.092	273.272	296.648

**Table 5:** The basic statistics of using a PFNF algorithm on Douban datasets.

Matrics	Before using PFNF algorithm	After using a PFNF algorithm			
		JC	CN	PA	RAI
Graph type	Undirected	Undirected	Undirected	Undirected	Undirected
No. of users (nodes)	1384	1384	1384	1384	1384
No. of relations (links)	25361	306900	205300	597689	597839
Network density	0.026	0.321	0.182	0.625	0.625
Average degree	36.649	443.497	148.231	863.712	863.929

### 3.2 The Power of Utilizing Explicit and Implicit Friendships

**Fig 4:**
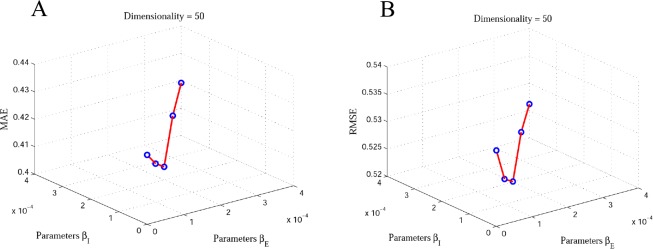
The impact of explicit and implicit friendships on Last.fm dataset with 80% of rating data. A: the MAE 3D plot of impact of explicit and implicit friendships; B: the RMAE 3D plot of impact of explicit and implicit friendships.

**Fig 5:**
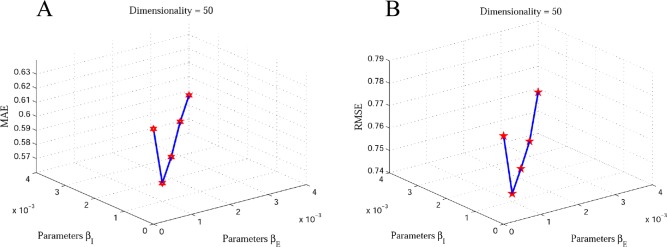
The impact of explicit and implicit friendships on Doudan dataset with 80% of rating data. A: the MAE 3D plot of impact of explicit and implicit friendships; B: the RMAE 3D plot of impact of explicit and implicit friendships.

In this paper, we incorporate two types of social information, explicit and implicit friendships, to generate the recommendations. In the objective function of our proposed model, there are two important parameters *β*_*E*_ and *β*_*I*_, which play key roles in controlling the contribution of the SR. They control how much the proposed method should incorporate the explicit and implicit friendships. If the two parameters are equal to 0, we only use the user-item rating matrix for matrix factorization to generate the final recommendations. If the parameter *β*_*I*_ equals 0, that means we use the user-item rating matrix and explicit and implicit friendships information for matrix factorization to generate the final recommendations. We note that the implicit friendship network is more dense than the explicit friendship network, as can be seen in Tables 4 and 5. Therefore, we should find the suitable values for the parameters *β*_*E*_ and *β*_*I*_ to balance. Hence, for simplifying our learning model we make *β*_*E*_ and *β*_*I*_ equal the same value. We tune the parameters *β*_*E*_ and *β*_*I*_ for our proposed method EISR by grid search (*β*_*E*_ and *β*_*I*_ = 0, 0.0001, 0.0002, 0.0003, 0.0004) and (*β*_*E*_ and *β*_*I*_ = 0, 0.001, 0.002, 0.003, 0.004) on Last.fm and Douban datasets respectively to reach the best performance. Figs 4 and 5 depict the impact of *β*_*E*_ and *β*_*I*_ on recommendation accuracy. This clearly shows that the MAE and RMSE values decrease when we choose (*β*_*E*_, *β*_*I*_ = 0.0002) and (*β*_*E*_, *β*_*I*_ = 0.001) on Last.fm and Douban datasets respectively. We can observe that incorporating explicit and implicit friendship information on our method demonstrates that the social friendship factors are more effective for improving the recommendation accuracy.

### 3.3 Impact of Feature Dimensionality *K* on the Result

**Fig 6:**
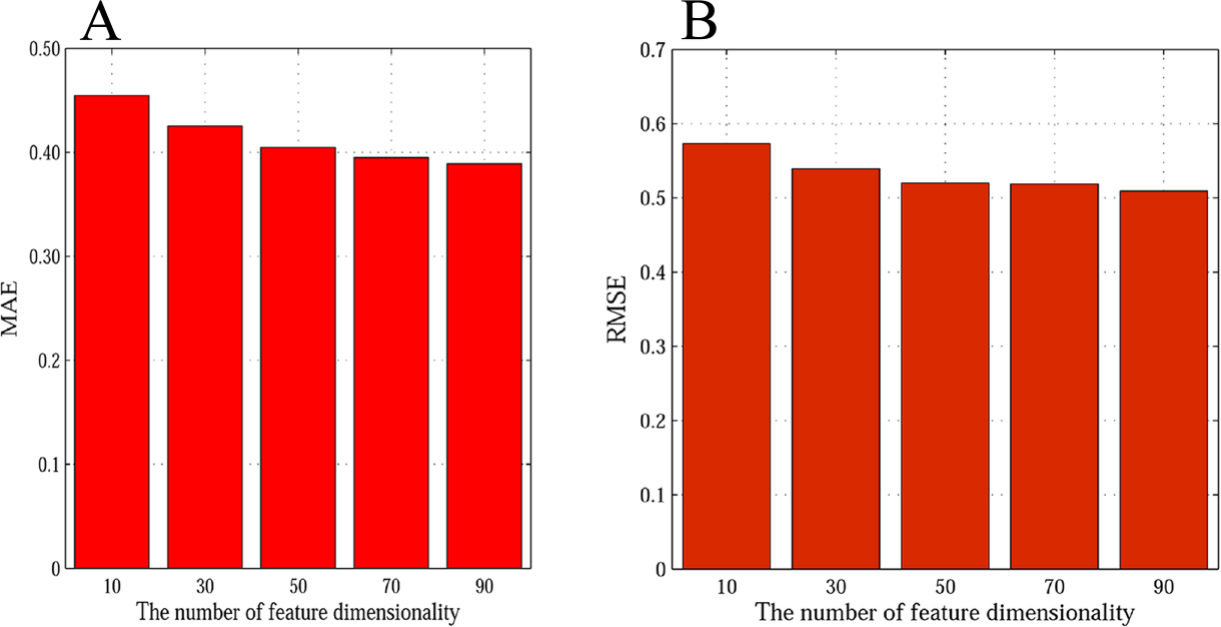
The impact of feature dimension on Last.fm dataset with 80% of rating data. A: the MAE histogram of impact of feature dimension; B: the RMAE histogram of impact of feature dimension.

**Fig 7:**
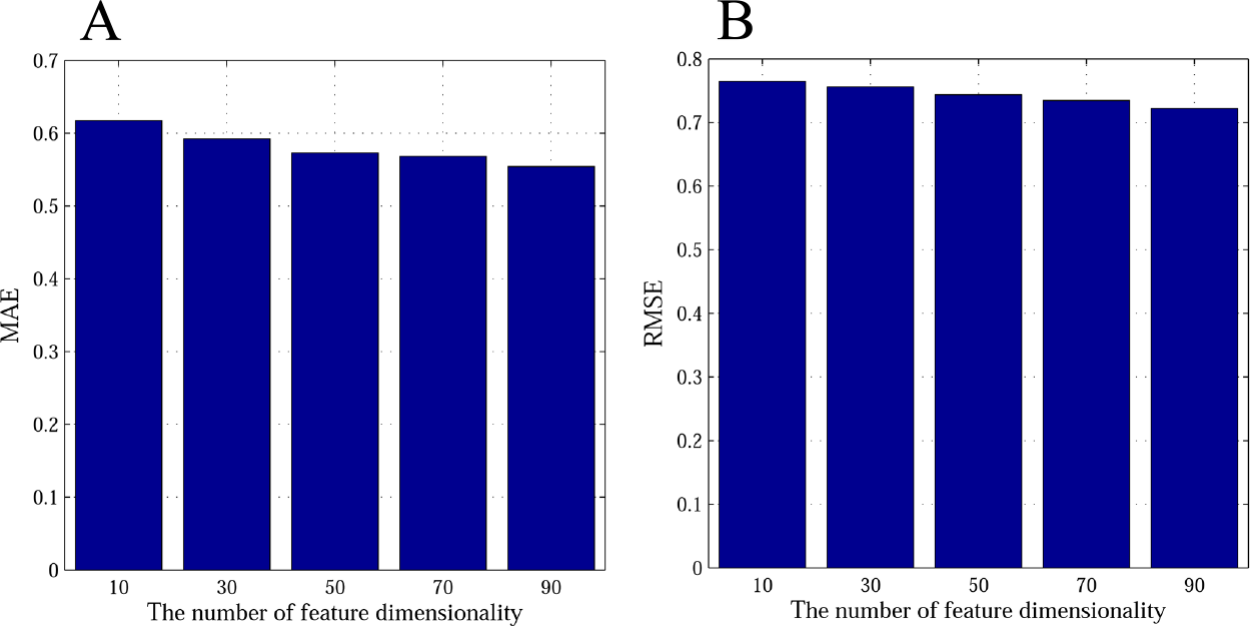
The impact of feature dimension on Doudan dataset with 80% of rating data. A: the MAE histogram of impact of feature dimension; B: the RMAE histogram of impact of feature dimension.

In addition to the power of the explicit and implicit friendship parameters to improve accuracy, feature dimensionality plays an important role in our proposed method EISR. We now turn to analyzing the effect of changing the dimension on the final recommendation. A different number of feature dimensions on prediction error are observed in this experiment. Figs 6 and 7 depict the prediction accuracy for the different values of *K*. From the results, we observe that the MAE and RMSE values decrease when *K* increases, but we can also see that when *K* > 50 on Last.fm and Douban, the MAE and RMSE decrease rather slowly. Considering the time efficiency, we choose *K* = 50 on Last.fm and Douban as the latent features dimension in our experiments. We can see that the increase in the feature dimensionality *K* improves the prediction accuracy of EISR. This may indicate that our proposed method is more stable in a situation with high dimensions.

## Conclusion

To deal with the sparsity problem, in this paper, we investigate the ability of adopting different social network relation types. We propose the PFNF algorithm to extract the hidden social relation based on the link prediction methods. Furthermore, we propose an explicit and implicit social relation (EISR) method based on the social network information, which considers the effects of both explicit friendship and implicit friendship. We integrate them into PMF in order to obtain better recommendation results. The experimental results on two real-world datasets, Last.fm and Douban, show that our method EISR obtains more accurate recommendation results than state-of-the-art recommendation methods, such as PMF, SR, HeatS and HP. In addition, our PFNF algorithm that is based on Resource allocation index (RAI) similarity metric for extracting the implicit friendship is better predictive than the rest of the link prediction methods.

Notably, with the explosion of Web 2.0 applications, social network users generate large volumes of information, which opens new doors for the social recommendation systems to exploit. However, in this article we only provide a simple start for utilizing social network topology to extract the hidden relation. While an implicit friendship may be based on several user actions (likes, comments, opinions and tags) the activities between users in the social network remains open for future study. But one challenge is that, as far as we know, there is no publicly available dataset that includes user activities’ information. Furthermore, since we only use the classical measurements to compute the similarity between users, how to design a more effective similarity function is another research direction. Also, our proposed method is general since it can easily incorporate different social network information, therefore, we plan to investigate whether social tagging is useful for our proposed method to improve the recommendation accuracy. Finally, as a continuation of this work, we plan to extend our proposed method (EISR) by using additional link prediction methods, to assess the results if these methods are used.

## Supporting Information

S1 Data Last.fm Dataset. (RAR)

S2 Data Douban Dataset. (RAR)
